# Sugarcane multitrophic interactions: Integrating belowground and aboveground organisms

**DOI:** 10.1590/1678-4685-GMB-2022-0163

**Published:** 2022-12-09

**Authors:** Diego Z. Gallan, Augusto B. Penteriche, Maressa O. Henrique, Marcio C. Silva-Filho

**Affiliations:** 1 Universidade de São Paulo, Escola Superior de Agricultura Luiz de Queiroz, Departamento de Genética, Piracicaba, SP, Brazil.

**Keywords:** Plant defense mechanisms, insect behavioral manipulation, plant-insect-fungus-virus-bacterium interactions, direct and indirect defense, functional diversity

## Abstract

Sugarcane is a crop of major importance used mainly for sugar and biofuel production, and many additional applications of its byproducts are being developed. Sugarcane cultivation is plagued by many insect pests and pathogens that reduce sugarcane yields overall. Recently emerging studies have shown complex multitrophic interactions in cultivated areas, such as the induction of sugarcane defense-related proteins by insect herbivory that function against fungal pathogens that commonly appear after mechanical damage. Fungi and viruses infecting sugarcane also modulate insect behavior, for example, by causing changes in volatile compounds responsible for insect attraction or repelling natural vector enemies via a mechanism that increases pathogen dissemination from infected plants to healthy ones. Interestingly, the fungus *Fusarium verticillioides* is capable of being vertically transmitted to insect offspring, ensuring its persistence in the field. Understanding multitrophic complexes is important to develop better strategies for controlling pathosystems affecting sugarcane and other important crops and highlights the importance of not only studying binary interactions but also adding as many variables as possible to effectively translate laboratory research to real-life conditions.

## Introduction

Sugarcane is an allogamous plant belonging to the Poaceae family and the genus *Saccharum* (Morais et al., 2015). It was originally from tropical regions of South and Southeast Asia and was introduced into the Americas during the second expedition of Christopher Columbus in mid-1496 and into Brazil in 1502 by Martim Afonso de Souza, with the introduction of seedlings from the Madeira Island (Daniels and Roach, 1987; Cesnik, 2007). Currently, sugarcane hybrids are grown all around the world, as they show superior agronomic characteristics to their parents (Bastos et al., 2003; Cesnik, 2007). The genetic basis of modern varieties comes from crosses between six sugarcane species resulting in interspecific hybrids; however, the most commonly used hybrids are originate from *S*. *officinarum* and *S*. *spontaneum* (Irvine, 1999; Li et al., 2022). These species display contrasting characteristics, with *S*. *officinarum* being characterized by a high sugar content, thick stem, low fiber content and low disease resistance, whereas *S*. *spontaneum* has a low sugar content, thin stem, high fiber content and high resistance to biotic and abiotic stresses (Sreenivasan and Ahloowalia, 1987; Singh et al., 2010). The combination of desired traits from these crosses results in plants with a high sugar content, vegetative vigor and resistance to diseases (Irvine, 1999).

The sugarcane crop is of great importance in tropical and subtropical regions of the world, being planted in more than 100 countries and covering approximately 24 million hectares (FAO, 2020). In Brazil, approximately 9 million hectares are cultivated with sugarcane (Conab, 2022), accounting for 43% of all global production, making Brazil the world’s largest producer of sugarcane, followed by India (17%) and China (7%) (Gallan, 2019; FAO, 2020). The sugarcane yield accounts for more than 70% of total sugar production worldwide, and it is one of the most efficient biological raw materials for ethanol, butanol and diesel production. New applications of industrial sugarcane residues, such as the use of bagasse for cellulose fiber extraction and second-generation biofuel production, have been on the rise recently, and other sugarcane byproducts include acetic acid, plywood, field fertilizers, and culture substrates for fruit tree seedlings (Neves et al., 2016; Sindhu et al., 2016; Jansen et al., 2017; Rahman et al., 2019; Lu et al., 2020; Budeguer et al., 2021; Mahmud and Anannya, 2021).

**Figure 1 -:**
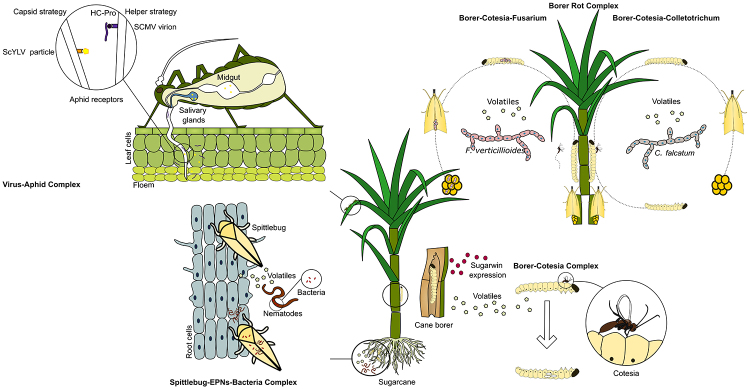
Multitrophic interactions in sugarcane. In the Virus-Aphid Complex, viral pathogens such as sugarcane mosaic virus (SCMV) and *yellow leaf virus* (ScYLV) are transmitted through different molecular strategies by aphids feeding on sugarcane leaves. The Spittlebug-EPNs-Bacteria Complex act in root tissue damaged by herbivory which release volatiles organic compounds (VOCs) attractive to nematodes bearing entomopathogenic bacteria lethal to the spittlebug, and released after the nematode enters its host. In the Borer-Rot Complex, *D*. *saccharalis* stem herbivory increases the expression of *Sugarwins*, wound-inducible proteins shown to act selectively against fungal pathogens, but not cause any damage to the insect. Rot causing pathogen *Fusarium verticillioides* is capable of drastically increase its dissemination by changing VOC profile during infection, manipulating borer and moth behaviors and being transmitted vertically to *D*. *saccharalis* offspring. *Colletotrichum falcatum* also takes advantage of VOC profile alteration during infection and increase its dissemination, as vectors present increased preference to infected plants. The Borer-Cotesia Complex is widely explored in biocontrol strategies as *C*. *flavipes* is involved with parasitism of *D*. *saccharalis* borers, and is attracted by VOCs from insect herbivory and feces. In addition, the VOC profile change caused by the presence of *F*. *verticillioides* decreases *C*. *flavipes* attraction to the plant and, consequently, to its host.

In the field, sugarcane plants are exposed to a myriad of biological interactions that may occur all at once or in different combinations (Figure 1), to which the plant responds by modulating a vast repertoire of defense-related genes to achieve healthy development and a good agronomic yield (Lo Presti et al., 2015; Souza et al., 2017; Sathyabhama et al., 2022). Plant defenses include the accumulation of secondary metabolites that can act as signals for the upregulation of defense response genes, such as wound-induced or pathogenesis-related protein-encoding genes, or even as volatiles or exudates that attract antagonists of herbivores and pathogens (Souza *et al*., 2017; Zhang et al., 2017; Erb et al., 2021; Divekar et al., 2022).

Herbivorous insects and phytopathogens lead to the estimated loss of 10-15% of the world’s major crops and losses of hundreds of billions of dollars (Cheavegatti-Gianotto et al., 2011; Peng et al., 2021). Currently, the main limiting factor in sugarcane production is the damage caused by the borer *Diatraea saccharalis*, although other insects, such as *Mahanarva fimbriolata* and many aphid species, can also cause losses by reducing plant weight gain, acting as vectors of phytopathogens and their respective diseases or killing the plant entirely (Garcia et al., 2007a; Sandoval and Senô, 2010; Srikanth et al., 2011).

Among all major crop diseases, 70-80% are caused by pathogenic fungi, and viruses, bacteria and oomycetes account for the remainder (Li et al., 2017; Peng et al., 2021). In sugarcane, more than 240 diseases have been described, among which red rot and Fusarium stem rot, leaf scald, ratoon stunting, mosaic, red streak, brown spot, brown rust and orange rust are the main concerns in Brazil (Saumtally et al., 2000; Cheavegatti-Gianotto et al., 2011; Gonçalves et al., 2012; Srivastava and Rai, 2012; Chaves et al., 2013; Morais et al., 2015; Sharma et al., 2017; Urashima et al., 2017; Borella et al., 2021; Costa et al., 2021). Some diseases may also involve multiple organisms as part of a complex multitrophic system, which will be further investigated in this review.

Rot-causing fungi such as *Fusarium verticillioides* and *Colletotrichum falcatum* are ubiquitous in sugarcane fields and are intimately associated with the borer *D*. *saccharalis*; these species cause devastating damage to plantations worldwide when coassociated and are known as the borer-rot complex (Franco et al., 2017; Arora and Malik, 2021). Control strategies include the release of *Cotesia flavipes*, a natural enemy of the borer, in *D*. *saccharalis-*infested fields; this strategy relies on another multitrophic interaction, herein referred to as the Borer-Cotesia-Fusarium complex, as the fungus has been described as being capable of influencing insect behavior and interacting with the Borer-Cotesia complex (Peñaflor and Bento, 2019; Franco *et al*., 2021). Finally, sugarcane-infecting viruses use sap-feeding aphids as vectors for their dissemination and are involved in a complex interaction that also involves insect behavioral manipulation and molecular mechanisms that interact with both the plant and vector, herein referred to as the virus-aphid complex (Lefeuvre et al., 2019; Akbar et al., 2020).

This review comprises the most recent information on multitrophic interactions relevant to sugarcane production and highlights the importance of studying and understanding nonbinary pathosystems to help develop disease control strategies. Furthermore, this review focuses on the molecular mechanisms already described in multitrophic complexes associated with sugarcane, providing substantial evidence that insect behavioral manipulation plays a major role in pathogen dissemination in a process that not only takes advantage of the damage caused by insect herbivory but has also evolved molecular strategies to promote a mechanism of increased transmission.

## Borer-Rot Complex

One of the best-studied multitrophic interactions in sugarcane is that of the borer-rot complex, representing an interaction between the Fusarium stem rot causal agent *F*. *verticillioides* and the red rot pathogen *Colletotrichum falcatum* with the sugarcane borer *Diatraea saccharalis*. Both pathogens are commonly associated with the presence of the borer in sugarcane crop fields and amplify its damage output and dissemination when they cooccur in this pathosystem (Dinardo-Miranda et al., 2008; Medeiros et al., 2012; Franco et al., 2017). The borer-rot complex is broadly distributed around the world, and in Brazil, there are reports of large losses due to rot when it is associated with a high intensity of *D*. *saccharalis* infestation (Amorim et al., 2011; Leal et al., 2013; Vargas et al., 2015).

*Diatraea saccharalis* (F.) (Lepidoptera: Crambidae) is one of the main pests of sugarcane and is widely distributed in sugarcane regions worldwide. In Brazil, it can lead to significant losses in production, and the main focus of this problem is the Southeast region (Botelho et al., 1999; Francischini et al., 2017). It is also considered a pest that impacts corn, rice, sorghum, and Sudan grass (Sidhu et al., 2013, 2014; Grimi et al., 2018; Araujo et al., 2019). Overall, this pest is difficult to control due to its cryptic habitat, as it lodges itself in galleries inside sugarcane stems, resulting in small circular holes that are difficult to spot (Malan and Hatting, 2015).

The damage caused by these caterpillars can be directly caused by their feeding on stem tissue, resulting in the formation of galleries that structurally weaken the plant, leading to weight loss and death of the buds. In new canes, it can affect shoot growth (causing a so-called “dead heart”) and lead to the formation of lateral shoots and aerial rooting, drastically affecting productivity (Gallo et al., 1988; Vargas et al., 2015). The borer also causes serious damage to the final products of the sugarcane industry, reducing the fermentative efficiency of molasses for ethanol production due to the formation of phenolic compounds and volatile organic acids by the plant (Lopes et al., 2016; Solomon, 2016). Indirect losses caused by pathogens such as *C*. *falcatum* and *F*. *verticillioides* are the most significant effects and are associated with borer presence, which increases pathogenicity and dissemination efficiency. Under some field conditions, up to 100% crop area infestation may occur, with major impacts on crop yield (Mahlanza, 2012; Vilela et al., 2017).

*Fusarium verticillioides* (Sacc.) Nirenberg (Nirenberg and O’donnell, 1998) (Holomorph: *Gibberella moniliformis* Wineland; synonym *F*. *moniliforme*) is a worldwide-dispersed fungal phytopathogen of great economic importance that infects both monocotyledons and dicotyledons. The affected crops include sugarcane (Ogunwolu et al., 1991; Wang et al., 2020), maize (Oren et al., 2003; Alberts et al., 2016), rice (Desjardins et al., 2000), wheat (Desjardins and Proctor, 2007), banana (Anthony et al., 2004), asparagus (Corpas-Hervias et al., 2006), and sorghum (Tesso et al., 2004), and the fungus is associated with various diseases, such as stem and root rot, fusariosis, seedling blight and pokkah boeng (Tiwari et al., 2020; Nagraj et al., 2021). It is a known mycotoxigenic organism that produces fumonisin B1-4, highly stable molecules involved in numerous health-related issues and a major problem in cereal production (Nelson et al., 1994; Bacon et al., 2008; Nagraj *et al*., 2021).

In sugarcane, *F*. *verticillioides* is one of the main causal agents of pokkah boeng disease (Lin et al., 2016; Tiwari et al., 2020; Viswanathan, 2020), which leads to symptoms ranging from twisted and shortened leaves to chlorotic striped areas that develop into necrotic rotted tissue in stems and leaves (Anthony et al., 2004; Tiwari *et al*., 2017, 2020), first observed by Walker and Went (1896) in Java. Nonetheless, the most detrimental damage is caused by the infection of the apical region, leading to top rot damage and the loss of the entire plant (Lin *et al*., 2016). Generally a hot humid and rainy season favors the disease and early stages of sugarcane are more prone to disease development than the matured canes (Martin et al., 1989; Viswanathan, 2020).

When *F*. *verticillioides* infection occurs in the initial stages of sugarcane development, substantial problems such as poor growth of the root system, loss of vigor, root rot and damping off occur, and the physiological damage caused by this pathogen has been linked to total loss of large cultivated fields (Ogunwolu et al., 1991; Tokeshi, 1997; Hsuan et al., 2011; Dean et al., 2012; Matny, 2015; Viswanathan et al., 2017). However, if disease onset occurs in the final stages of plant development, its effects are usually of a lesser magnitude since the plant defenses against biotic stresses are already fully prepared. In addition, the plant can still be used for production, and if necessary, early harvesting can be carried out to avoid causing major problems, even though infection in these stages is rare (Debach and Rosen, 1991; Fracchia et al., 2014; Lacava and Azevedo, 2014).

To control this pathogen in sugarcane, the only approach developed to date is the use of resistant varieties, and there are no chemical products registered for *F*. *verticillioides* control (Wang et al., 2020). Producers who use intermediate or susceptible varieties can only prevent the infestation of this pathogen when operating in soils and environments that are highly suitable for the crop and, if possible, in regions where the pathogen is absent. In any situation outside of this scenario, the plant will manifest some stress symptoms of this disease (Campanhola et al., 1998; Pérez-Montaño et al., 2014). Resistant varieties, however, tend to eventually succumb to the disease, probably due to different environment conditions or the susceptibility to a different pathogen (Viswanathan, 2020).

*Colletotrichum falcatum* Went [*Glomerella tucumanensis* (Speg) Von Arx.] is the causal agent of red rot, one of the most damaging diseases in sugarcane. It mainly causes stem rot and leaf lesions, and can affect any development stage of the plant. Symptoms include reddish discoloration of stem and leaf tissue and appearance of white spots in the center, as well as drying of leaf and stem, death of new sprouts and formation of pith cavities filled with grey mycelia in later stages of disease development (Tokeshi, 1997; Franco et al., 2017). Red rot has been shown to co-occur with other sugarcane pathogens such as wilt pathogen *F*. *sacchari*, pineapple disease pathogen *Ceratocystis paradoxa* and stem rot pathogen *F*. *verticillioides*, with overlapping symptoms including drying, pith cavities formation and reddish to purple discoloration, although red rot can be easily distinguished by the reddening of internal tissue with white spots (Viswanathan, 2021). Colonization by these fungi disrupts water and nutrient transport in plants and causes tissue damage, as they can act as necrotrophic organisms, leading to reduced biomass and consequently reduced sugar and alcohol production (Narayanasamy, 2013; Sharma et al., 2017; Peñaflor and Bento, 2019). *C*. *falcatum* and *F*. *verticillioides* both cause the inversion of sucrose into glucose and levulose, which are noncrystallizable sugars that reduce sugar yields, and the presence of these organisms in sugarcane stems can lead to contamination and active competition with yeasts responsible for sugar fermentation (Dinardo-Miranda et al., 2008; Medeiros et al., 2012; Duraisam et al., 2017; Peñaflor and Bento, 2019).

The most effective method for preventing *C*. *falcatum* infection is again to use varieties that are resistant and/or adapted to the type of soil and typical climate of the region, reducing any stress to the plant as much as possible (Fávaro et al., 2012; Narayanasamy, 2013).

The caterpillar *D*. *saccharalis* provides an ideal environment for both *F*. *verticillioides* and *C*. *falcatum* and, once present in field crops, kills the plant tissue cells through the action of its digestive system and deposits the dead material in its path left in the stem, favoring the occupation of these pathogens (Tokeshi, 1997; Matsuoka, 2013). There is no evidence of the presence of *F*. *verticillioides* in sugarcane fields where the pest *D*. *saccharalis* is not also present in Brazil; however, *C*. *falcatum* has been reported in the absence of the insect in other countries, such as India, Australia, Thailand, Fiji, and the United States (Singh, 1998; Singh *et al*., 2010; Gallan, 2019; Da Silva *et al*., 2021).

Until recently, it was believed that *F*. *verticillioides* and *C*. *falcatum* were opportunistic fungal pathogens that accessed plants exclusively through openings left following borer herbivory (Ogunwolu et al., 1991; Franco et al., 2017). However, Franco *et al*. (2021) showed that *F*. *verticillioides* is capable of modulating the profile of volatile organic compounds (VOCs) in the plant, thereby causing changes in insect behavior and being transmitted vertically through females to their offspring. Adult insects infected with the fungus prefer to lay their eggs in healthy plants, while noninfected insects prefer *F*. *verticillioides*-infected plants for feeding and oviposition. Caterpillars also prefer a fungus-infected diet in olfactory dual choice assays (Franco *et al*., 2021). Similar behavior has been shown in *C falcatum*, although to a lesser extent as the fungus is not transmitted to the next generation (Franco *et al*., 2022). This represents a major change in the view of the fungal role in the borer-rot complex interaction, as both *F*. *verticillioides* and *C*. *falcatum* are able to significantly increase their dissemination by increasing insect attraction and offspring transmission in *D*. *saccharalis* (Franco *et al*., 2021; Franco *et al*., 2022). As the two fungi can coinfect sugarcane and be transmitted by *D*. *saccharalis*, it is possible that the VOC profile modulation induced by one fungus could increase the dissemination of the other as well.

This multitrophic interaction seems to be tightly interconnected up to the molecular level. The herbivory of *D*. *saccharalis* in sugarcane stems increases the expression of defense-related genes, including those encoding pathogenesis-related proteins (PRs) such as SUGARWIN2, a PR-4 that has a direct impact on *F*. *verticillioides* and *C*. *falcatum* survival, leading to fracture points in the hyphae, changes in morphology and extensive intracellular leakage but causing no damage to the insect (Medeiros et al., 2012; Franco et al., 2017, 2019). This effect could be explained by the chitosanase activity that is shared by SUGARWIN2 and its homolog SUGARWIN1, as they are able to cleave one of the main components of the fungal cell wall (Franco *et al*., 2019; Maia et al., 2021). The expression of SUGARWIN1 is also increased under these conditions, and its RNAse and chitinase activities, which are not observed in SUGARWIN2, suggest other roles in plant defense. Nevertheless, this antimicrobial activity is pathogen selective, as it does not cause any damage to *Aspergillus nidulans* or *Saccharomyces cerevisiae*, indicating a complex level of evolutionary specificity (Medeiros *et al*., 2012; Franco *et al*., 2014). Both proteins are targeted to the extracellular space and present a typical signal peptide in their sequence causing them to accumulate in wounded areas in a mechanism for containing invading microorganisms that closely follow insect herbivory (Medeiros *et al*., 2012; Franco *et al*., 2019; Maia *et al*., 2021). Varieties with higher SUGARWIN expression also present increased tolerance to *C*. *falcatum* and *F*. *verticillioides* infection, highlighting an important mycoprotective role of these proteins against pathogen interactions in sugarcane (Franco *et al*., 2019; Javed et al., 2019).

## Borer-Cotesia-Fusarium Complex

One of the most effective ways to control sugarcane borer populations is to release the biological control agent *Cotesia flavipes* Cameron (Hymenoptera: Braconidae), an exotic parasitoid of generalist larvae introduced in Brazil in the 1970s (Legaspi et al., 1997; Botelho and Macedo, 2002; Greathead and Neuenschwander, 2003; Frank and Mccoy, 2007). This control strategy was so efficient that in the 1980s, the population of the borer decreased to only 2% of their previous sizes, which was very favorable for the sugar-alcohol sector (Macedo *et al*., 1984; Consoli et al., 2001).

This form of biological control continues to the present day, and these parasitoids are currently released over almost 3.5 million hectares of sugarcane, corresponding to 90% of the total cultivated area (Parra, 2014; Parra and Coelho, 2022). Due to the low cost, easy acquisition and operation of this method, in addition to its ability to reduce losses caused by the borer, it results in cost savings in the industry related to the purchase and application of pesticides and the related labor required (Teran and Novaretti, 1980; Overholt et al., 1997; Aya et al., 2017).

Biological control is achieved because the parasitoid only completes its life cycle when associated with a borer, shaping the borer-Cotesia complex. The wasp looks for a suitable host and lays its eggs inside the borer caterpillar; the wasp larvae then develop after hatching by feeding on *D*. *saccharalis* larvae from the inside until the host eventually dies from exhaustion (Godfray, 1994; Rogers, 2003). After reaching the complete larval stage, the caterpillars of *C*. *flavipes* migrate outside of the *Diatraea* body*,* and the pupal stage begins, which is identified by linked white cocoons, forming a white “mass” (Moutia and Courtois, 1952; Parra, 2012). After several days, adults emerge and typically mate soon after birth, causing the cycle to effectively start again (Moutia and Courtois, 1952; Wiedenmann et al., 1992; Overholt et al., 1994; Singer and Parmesan, 2010).

*C*. *flavipes* females use olfactory stimuli to locate host-infested plants (Potting et al., 1995; Xiaoyi and Zhongqi, 2008). The main source of volatiles in the plant-host complex is the stem injured by the caterpillar, including the feces and the regurgitated material produced by the caterpillar (Potting *et al*., 1997; Usha Rani, 2014; Kant et al., 2015). However, the production of volatile substances attractive to parasitoids is not restricted to the infested part of the plant and can also occur systematically throughout the plant (Van Leerdam et al., 1985; Potting *et al*., 1995; Potting *et al*., 1997).

For the natural enemy to effectively find its host, it is necessary for a mixture of volatile compounds to be present, and it is very difficult to precisely detect the identities and quantities of these compounds (Mccormick et al., 2012; Tasin et al., 2012; Clavijo Mccormick et al., 2014). Important compounds that may be involved in this Borer-Cotesia complex include (Z)-3-hexenyl acetate, (E)-4,8-dimethyl-1,3,7-nonatriene, heptanal, and (E)-β-farnesene; however, due to the complexity of these interactions, there are still discussions and studies concerning their definition (Ngi-Song et al., 2000; Bruce et al., 2010).

Fungal phytopathogens induce changes in the metabolite profiles of plants and the degree of defense against herbivorous insects (Ako et al., 2003; Poelman et al., 2008; Tack et al., 2012; Gols, 2014). Fungal infection in plants can also affect the natural enemies of herbivores, which are guided by chemical signals from plants (Cardoza et al., 2002; Piesik et al., 2009; Desurmont et al., 2016; Eberl et al., 2018). For example, when *F*. *verticillioides* (Peñaflor and Bento, 2019) interacts with the Borer-Cotesia Complex, it modifies the entire mechanism and gives rise to a new Borer-Cotesia-Fusarium Complex.

When sugarcane borer attack is associated with Fusarium stem rot infection, it induces the production of a volatile mixture containing the typical fungal volatile 1-octen-3-ol (Inamdar et al., 2013), 2-β-pinene and 6-methyl-5-hepten-2-one, differing from the mixture observed in the absence of the pathogen, in addition to altering the composition of herbivore-induced plant volatiles and decreasing the amounts of α-pinene, α-limonene and 2-dodecen-1-al (Peñaflor and Bento, 2019). Therefore, the release of a different volatile mixture by the plants in conjunction with Fusarium infection causes a change in parasitoid behavior, in which the parasitoids prefer volatiles released by healthy plants attacked the borer over plants of the same condition that are also infected with *F*. *verticillioides* (Peñaflor and Bento, 2019).

Thus, the presence of *F*. *verticillioides* prevents the detection of the borer by *C*. *flavipes*, diminishing its biological control efficiency (Peñaflor and Bento, 2019). This fact, together with both recently published and previously reported studies in which the occurrence of vertical transfer and insect behavioral manipulation by this pathogen were identified (Franco et al., 2021), makes it possible to infer the presence of a complex mechanism of interaction both affecting and evolving within this multitrophic system.

## Spittlebug-EPN-Bacteria Complex

The sugarcane spittlebug *Mahanarva fimbriolata* (Hemiptera: Cercopidae) was first described as belonging to the genus Monecphora in 1854 (Guagliumi, 1970). In 1968, the species was transferred to the genus to which it belongs today based on considering the morphological characteristics of the male genitalia (Fennah, 1968).

This species is hemimetabolous, passing through egg, nymph, and adult stages (Terán, 1987). The newly hatched nymphs have a size of approximately 1 mm and, after four ecdyses, reach a size of up to 10 mm before undergoing the last ecdysis and entering the adult stage (Mendonça et al., 1996).

Nymphs preferentially attack the roots of host plants, mainly consisting of sugarcane, causing an effect known as a “physiological disorder” that prevents the flow of water and nutrients, leads to root necrosis and favors the entry of pathogenic fungi (Garcia et al., 2007b; Tonelli et al., 2016). The adults live in the aerial part of the plant and feed by sucking sap from the (preferably apical) leaves and the green parts of the stem (Guagliumi, 1970; Botelho et al., 1976; Akbar, 2009). Mating occurs soon after the emergence of the adult, regardless of the time of day (Garcia *et al*., 2011). The eggs are deposited close to the roots in clumps in the soil near the culm and particularly in the dry sheaths (Guagliumi, 1970; Pires, 1998).

With the expansion of areas where sugarcane is harvested without fire, the spittlebug has become a very important pest, as the straw remaining in the area provides an ideal microclimate for its development and dissemination (Ma et al., 2014; Carvalho et al., 2017; White, 2019). An effective form of biological control is to use entomopathogenic nematodes (EPNs) (Batista and Auad, 2010; Batista *et al*., 2014; Tonelli et al., 2016).

Nematodes (also known as roundworms) are nonsegmented organisms that belong to the phylum Nemata, one of the most numerous groups on the planet (Kaya and Stock, 1997; Paily et al., 2009). Some nematodes have the ability to cause the death of insects, which is why they are categorized as entomopathogenic (Dillman et al., 2012). The main species with an entomopathogenic capacity are found in the genera *Steinernema*, *Neosteinernema* and *Heterorhabditis* (Hominick et al., 1996; Burnell and Stock, 2000; Hazir et al., 2004; Campos-Herrera et al., 2012). The life cycle of entomopathogenic nematodes includes egg, juvenile and adult stages, among which infectious juveniles are capable of infecting the host (Ebssa et al., 2004; Noguez et al., 2012).

On the ground, infectious juveniles search for a host to penetrate and then migrate to the hemocoel via body openings (mouth, spiracles, and anal and genital pores) and cuticles (Poinar Jr, 1990; Kaya and Stock, 1997; Singha et al., 2022). The digestive tract of EPNs contains symbiotic bacteria whose metabolism and growth remain in a controlled state until an insect host is found (Ehlers, 2001; Ciche et al., 2006; Lewis et al., 2006; Stock and Blair, 2008; Crawford et al., 2010). There is great specificity in the symbiosis between the bacterium and the nematodes; for example, in nematodes of the *Heterorhabditis* genus, only bacteria of the genus *Photorhabdus* are found, while in nematodes of the genus *Steinernema*, only bacteria of the *Xenorhabdus* genus are found (Boemare, 2002; Adams et al., 2006; Sajnaga and Kazimierczak, 2020).

As soon as the nematodes reach the insect’s hemocoel, the infectious juveniles release the bacteria that they carry, initiating an infection that can lead to the death of the insect within 24 to 48 hours (Kaya and Stock, 1997; Vega and Kaya, 2012). Inside the insect, the nematodes feed, develop, mate and reproduce for multiple generations before the infectious juveniles break out of the host’s corpse and enter the environment (Kaya and Stock, 1997; Bohlmann, 2015).

The behavior of EPNs is coordinated and defined by the integration of many external stimuli, such as light, temperature, and the levels of chemical compounds, humidity, and carbon dioxide, which are sensed by cuticular and internal organs. Through this mechanism, EPNs utilize volatiles from roots damaged by herbivores to search for hosts (Burr and Robinson, 2004; Riga et al., 2004; Dillman et al., 2012).

In research related to better understanding the interaction mechanism involved in the spittlebug-EPN-bacteria complex, it has been presumed that the occupation of the same location by sugarcane spittlebug nymphs sucking xylem and phloem for 30 to 40 days would mean that nymphs are prey easily found by natural enemies (Pathak and Khan, 1994; Garcia et al., 2007b). Tonelli et al. (2016) observed a lower emission of volatiles from roots damaged by the spittlebug, which could be an adaptive strategy for making them less detectable, thus reducing their chance of being found by natural enemies. However, the results showed that EPNs are still oriented toward roots damaged by the insect, despite the reduced emission of 11 components, among which dihydromyrcenol and β-isomethyl ionone presented the largest reductions. The authors suggest that more studies are needed to fully understand this interaction and define the key compounds involved.

In the spittlebug-EPN-bacteria complex, several evolutionary hypotheses related to the defense and propagation mechanisms of these organisms can be feasibly proposed. This complex interaction is an example of how intrinsically related biological systems are outside of laboratory conditions and highlights the importance of understanding the many variables involved in multitrophic interactions to improve crop production efficiency. Here, we see that the feeding on sugarcane plants by herbivores such as spittlebugs leads to the activation of plant defense mechanisms that may be responsible for attracting the insect’s natural EPN enemies (Grunseich, 2021). In this specific interaction, the EPNs present an evolutionary advantage by carrying bacteria in a state of “hibernation”, which can increase spittlebug mortality and improve EPN survival; in turn, the bacteria obtain an advantage in reaching their host guided by the nematode’s locomotion and sensing mechanisms.

## Virus-Aphid Complex

Plant viruses are responsible for approximately 50 billion euros of economic losses around the world (Pallás et al., 2018), and more than 1000 species infecting cultivated plants have been described (Rao and Reddy, 2020). In Brazil, there are 213 cataloged virus species recognized by the International Committee on Taxonomy of Viruses (ICTV) and six plant viroids (Kitajima, 2020). Sugarcane-infecting viruses include *Potyvirus* sugarcane mosaic virus (SCMV), *Poaceae* polerovirus sugarcane yellow leaf virus (ScYLV) and badnavirus sugar cane bacilliform virus (SCBV). The two main viruses infecting sugarcane are SCMV and ScYLV (Gonçalves et al., 2012), and mixed infections have been reported in the field (Madugula and Gali, 2018).

SCMV was first detected in sugarcane in 1919 and in maize in 1963 in the United States (Brandes, 1919; Janson and Ellett, 1963). It belongs to the sugarcane subgroup of mosaic viruses, together with maize dwarf mosaic virus (MDMV), Johnsongrass mosaic virus (JGMV), sorghum mosaic virus (SrMV), *Zea* mosaic virus (ZMV), *Pennisetum* mosaic virus (PeMV) and *Cocksfoot* strike virus (CSV), and it infects maize, sorghum, sugarcane and other poaceous species around the world (Wu et al., 2012). It is considered to be one of the 10 viruses causing the largest economic impact worldwide (Rybicki, 2015). In the beginning of the XX century, SCMV was introduced in Brazil and caused an epidemic in the sugar industry, mainly due to the susceptibility of varieties POJ 36, 213 and 218. The epidemic was later controlled by the substitution of these varieties for resistant hybrids (Koike and Gillaspie, 1989; Kitajima, 2020).

SCMV is a positive sense ssRNA, nonenveloped, monosegmented virus of the Potyviridae family; approximately 2000 protein monomers constitute its capsid, which are arranged in a helicoidal structure to form flexible virions 750 nm in length and 13 nm in height (Urcuqui-Inchima et al., 2001; Valli et al., 2015). The SCMV genome is approximately 10 kb, comprising one untranslated region (UTR) at each extremity and only one open reading frame (ORF). The ORF encodes a polyprotein of approximately 350 kDa, which is cleaved into the following 11 genic products, from the N- to C-termini: P1, Protein 1; HC-Pro, Helper component proteinase; P3, Protein 3; PIPO, Pretty interesting Potyviridae ORF; 6K1, Protein 6K1; CI, Cylindrical inclusion protein; 6K2, Protein 6K2; VPg, Viral protein genome-linked; NIa-Pro, Nuclear inclusion a protein; NIb-Pro, Nuclear inclusion b protein; and CP, Coat protein (Urcuqui-Inchima *et al*., 2001; Chung et al., 2008). The disease caused by SCMV affects photosynthesis directly due to chlorophyl destruction, leading to reductions in the total content of sugar and its crystallization rate, which may reduce the yield of sugarcane by up to 80% (Irvine, 1971; Koike and Gillaspie, 1989; Singh et al., 1997; Bagyalakshmi et al., 2019; Pan et al., 2021).

Sugarcane yellow leaf vírus (ScYLV) was first identified in Hawaii in 1989 and Brazil in 1990 and mainly constitutes a problem of certain susceptible sugarcane varieties, though it has also been described in species of Erianthus, barley [*Hordeum vulgare*], grain sorghum [*Sorghum bicolor*] and Columbus grass [*Sorghum almum*] (Schenck, 1997; Vega et al., 1997; Scagliusi and Lockhart, 2000; Schenck and Lehrer, 2000; Comstock et al., 2001; Bouallegue et al., 2014; Espinoza Delgado et al., 2016), It is a nonenveloped, (+)ssRNA, spherical, monosegmented virus of approximately 6 kb. It is also bound to a VPg at the 5’ extremity, including approximately 180 capsid proteins (Moonan et al., 2000; Smith et al., 2000; Fauquet et al., 2005). Its genome contains three UTRs: one 5’ UTR, one 3’ UTR and an intergenic UTR between ORF2 and ORF3 and is composed of six ORFs (0-5 from the 5’ to 3’), which encode the following proteins: protein P0 (ORF0); a polypeptide consisting of a genome-linked peptide (VPg) and a serine protease (ORF1); an RNA-dependent RNA polymerase (RdRp) translated from a −1 translational frame shift in ORF1 (ORF2); a viral coat protein (CP) (ORF3); a movement protein (MP) (ORF4); and a readthrough protein (RT) of the termination codon at the end of ORF3 fused to CP, translated from ORF5 (Moonan *et al*., 2000; Elsayed et al., 2011).

Yellow leaf (YL) caused by ScYLV, also known as yellowing, reaches phloem tissues and leaf veins to develop bright yellow coloration; this change is follows the chlorosis of the entire leaf blade, reducing cane growth, stem width and overall sucrose contents, leading to yield reductions of up to 50% (Vega et al., 1997; Lin et al., 2014; Holkar et al., 2020; Kitajima, 2020).

Plant viruses mostly depend on vectors for their survival and transmission (Raccah and Fereres, 2009). Both SCMV and ScYLV are naturally transmitted by aphids, and many aphid species have been reported to transmit viruses from diseased plants to healthy ones, including *Acyrthosiphon piston*, *Hysteroneura selariae*, *Myzus persicae*, *Rhopalosiphum maidis*, *Schizaphis grammum*, *Melanaphis sacchari*, *Ceratovacuna lanigera* and *Rhopalosiphum rufiabdominalis* (Komblas and Long, 1972; Holkar et al., 2020). All of these species act as vectors in sugarcane, among which *R*. *maidis* and *M*. *sacchari* are vectors of ScYLV (Lockhart and Cronjé, 2000), and *A*. *piston*, *H*. *selariae, M*. *persicae* and *S*. *grammum* are important aphid populations that can spread SCMV in sugarcane (Komblas and Long, 1972).

The transmission of plant viruses by insects can occur in a circulative or persistent manner or in a noncirculative, semipersistent or nonpersistent manner. In the first mode of transmission, ingested viral particles move through the intestinal epithelium to the hemocoel and then to the salivary gland (SG), crossing the SG membrane and being transmitted during feeding (Brault et al., 2007; Ammar et al., 2009; Raccah and Fereres, 2009; Dáder et al., 2017). The second mode of transmission involves a specific and reversible interaction between viral particles and the stylets or foreguts of aphids (Froissart et al., 2002; Uzest et al., 2007).

SCMV is transmitted nonpersistently (Xia et al., 1999). Both CP and HC-Pro have been described as being involved in its transmission, as CP can interact directly with vector receptors and ensure virus retention until it is released in the next host (Raccah and Fereres, 2009; Gadhave et al., 2020), and HC-Pro can form a molecular bridge between vector receptors and CP (Govier et al., 1977; Wang et al., 1998). These short-term, weak, reversible interactions render vector control strategies inefficient (Wu et al., 2012). SCMV forms genomic RNA replication sites in the cytoplasm and colocalizes with vesicles induced by 6K2-VPg-Pro proteins, which target multiple intracellular organelles, including the endoplasmic reticulum, Golgi, mitochondria and peroxisomes (Xie et al., 2021). In the cell-to-cell movement of potyviruses, PIPO interacts with P3, directing CI proteins to plasmodesmata to form a conical structure mediating intracellular virus movement (Chai et al., 2020). ScYLV can in turn be transmitted in both circulative and noncirculative modes (Schenck and Lehrer, 2000; Holkar et al., 2020), and the RT protein present in the capsid is responsible for virus transmission via aphids (Moonan et al., 2000; Smith et al., 2000).

Understanding the functions of proteins involved in these interactions as well as the molecular biology of plants, aphids and viruses is of the utmost importance for developing control strategies to reduce viral propagation and the damage caused by this multitrophic pathosystem in sugarcane. Interestingly, viruses transmitted by aphids seem to show a mechanism similar to that of the borer-rot complex that influences insect behavior; the effect of this mechanism is to make infected plants more attractive to sap-feeding insects or ensure that infected plants produce chemicals responsible for interfering with aphid behavior to increase virus dissemination, as described by the “vector manipulation hypothesis” (Blanc and Michalakis, 2016; Mauck, 2016; Dáder et al., 2017; Lefeuvre et al., 2019; Safari et al., 2019; Ziegler-Graff, 2020; Pan et al., 2021).

The expression of different genes is induced to combat pathogen infection in plants, and many of these genes have protein products. Several proteins involved in defense against biotic stresses in sugarcane have been described (Souza et al., 2017).

Infection by SCMV probably alters sugarcane physiology by increasing peroxidase activity in an attempt by the plant to respond to and inhibit virus development (Bhargava et al., 1970; Akbar et al., 2020). Comparative analyses show that sugarcane cultivars susceptible to SCMV exhibit the upregulation of transcripts related to sugar metabolism and transport relative to resistant cultivars, favoring viral replication (Akbar *et al*., 2021). Indeed, sugarcane leaves contaminated with SCMV exhibit superior sucrose accumulation to uninfected leaves, even though sucrose phosphate synthase (SPS) activity is reduced under these conditions (Addy et al., 2017).

Similarly, ScYLV infection leads to an increase in available soluble sugars (Gonçalves et al., 2005). ScYLV P0 has been described as targeting the plant argonaute 1 protein, which is involved in RNA interference (RNAi) processing and acts as a suppressor of RNA silencing (Baumberger et al., 2007; Csorba et al., 2010). Although this disease was reported over three decades ago, there are few studies on the interaction of ScYLV with its host (Holkar et al., 2020). However, there have been efforts to establish markers associated with resistance traits for use in sugarcane genetic improvement programs (Pimenta et al., 2021).

RNAi is used to produce virus-tolerant transgenic plants. Following this strategy, one approach to control SCMV is to generate transgenic sugarcane plants that express a short hairpin RNA (shRNA) that targets the sugarcane mosaic virus coat protein (CP) gene. Indeed, in the sugarcane cultivar SPF-232, the transgenic sgRNA4 line shows a reduction in the mRNA expression of CP-SCMV by up to 90%; thus, the plant is almost immune to SCMV infection (Aslam et al., 2018). Three-trophic-level interactions, such as the one described herein, are common in nature and in field crops; however, studies focusing on plant-virus-vector system dynamics have emerged only in the last decade, highlighting the importance of understanding the whole system over binary interactions alone (Pan et al., 2021).

## The complexity of multitrophic interactions

These three-part systems can continue to develop as more layers of multitrophism are added. For instance, the main control management strategy employed for *F*. *verticillioides* and *C*. *falcatum* rot is control of the borer *D*. *saccharalis* (Franco et al., 2014; Da Silva *et al*., 2021). Notably, biocontrol strategies such as the use of *Cotesia flavipes* to parasitize borer caterpillars represent a good alternative to the application of agrochemical substances (Molnár et al., 2016; Parra and Coelho, 2022). Recently, it was shown that sugarcane plants infected with *F*. *verticillioides* subjected to *D*. *saccharalis* attack release fewer VOCs that are attractive to *C*. *flavipes*, highlighting an indirect benefit of this interaction, even though the fungus seems to impair larval weight gain (Peñaflor and Bento, 2019; Franco *et al*., 2021). This weight reduction is hypothesized to be due to the production of toxins such as fumonisins that can also affect larval biology (Peñaflor and Bento, 2019); however, recent studies point to a possible new level of interaction, in which yeast and bacteria from the *D*. *saccharalis* microbiome seem to compete with *F*.*verticillioides* and *C*. *falcatum* and inhibit their growth under laboratory conditions (Da Silva *et al*., 2021).

There has been a recent focus on understanding the rhizosphere community composition, as it has a great impact on sugarcane development and resistance to pathogens such as *Ustilago*, *Fusarium* and *Colletotrichum* (Tayyab et al., 2022). Notably, it has been reported that *Pseudomonas* spp. mediate defense responses in sugarcane through the differential exudation of root phenolics, as well as inducing systemic resistance and antifungal activity against sugarcane pathogen *C*. *falcatum* in sugarcane stems (Shair et al., 2021), making it possible to speculate that another variable acts in this pathosystem. Interestingly, one of the strategies adopted in organic agriculture is the application of elicitors to cut the use of high-toxic microbicides, which in turn favors beneficial microorganisms (Zheng et al., 2020).

The spittlebug-EPN-bacteria complex shows highly conserved similarity to the borer-rot complex, as a “Trojan Horse” seems to be inserted into the interaction in both cases. *D*. *saccharalis* is to *F*. *verticillioides* what EPNs are to bacteria. As such, the complexity of the evolutionary mechanism of these complexes may still be far from fully defined and exploited.

In addition to plant-fungus-insect interactions, some fungal viruses are capable of replicating in plant cells, and some plant viruses are capable of replicating in fungal cells (Andika et al., 2017; Nerva et al., 2017; Mascia et al., 2019). However, there are fungal viruses distributed throughout the Fungi kingdom that can cause phenotypic changes in the host leading to reduced virulence (hypovirulence) (Nuss, 2005). Indeed, mycovirus infection in *F*. *sacchari* and *F*. *andiyazi*, pathogenic fungi that can cause pokkah boeng disease in sugarcane, might be associated with this hypovirulence, which is another factor that can interfere with rot complexes (Yao et al., 2020).

Plants are frequently attacked by viruses and their vectors in nature. However, the dynamics of the tripartite plant-virus-vector system, specifically regarding the impact of viral infection on plant-insect interactions, have just recently begun to emerge (Pan et al., 2021). In fact, the efforts of virome studies in which plant tissues, trapped or captured insects, and soil are collected over ecologically relevant areas are shifting away from individual host-virus-vector systems toward describing virus diversity and functions in the context of entire environments (Lefeuvre et al., 2019).

Plant viruses can interact with their insect vectors in a variety of ways, including nonpersistent and circulative modes of transmission. The interaction of a virus with its insect vector is characterized by molecular interactions between the virus and the insect, most typically mediated by proteins. Understanding how plant viruses interact with their insect vectors can contribute to the development of new strategies for protecting plants from infection by disrupting virus uptake and transmission (Dietzgen et al., 2016).

Novel methods such as RNAi and CRISPR gene editing are being used to develop long-term management alternatives. A successful attempt was recently made to use the CRISPR-Cas9 technique in the pea aphid *A*. *pisum* based on the microinjection of fertilized eggs with CRISPR-Cas9 components designed to edit Stylin-01, a cuticular protein gene (Le Trionnaire et al., 2019). However, it is unclear whether this alteration will affect CaMV and, by extension, potyviral transmission via aphids. If so, this knowledge could be extended to sugarcane and other crops taking part in plant-virus-vector complexes.

Plant viruses can influence the behavior of insect vectors both directly and indirectly by manipulating their plant hosts, resulting in increased transmission efficiency and dissemination (Blanc and Michalakis, 2016). The nonpersistently aphid-transmitted cucumber mosaic virus (CMV) can cause modifications in the host plant, such as the regulation of the jasmonic acid signaling system by the viral 2b protein, that can in turn modify the behavior of their insect vectors (Ziebell et al., 2011; Carmo-Sousa et al., 2014). The nuclear inclusion of a (NIa) protease protein of turnic mosaic potyvirus (TuMV) can manipulate host plant physiology to attract aphid vectors and to promote their reproduction (Casteel et al., 2014). The acquisition of Luteoviride, such as ScYLV, appears to alter the selection behavior of aphids so that they prefer uninfected plants, while nonviruliferous aphids tend to prefer virus-infected plants (Ingwell et al., 2012; Rajabaskar et al., 2014). Similar behavior has been reported in the borer-rot complex (Franco et al., 2021; Franco *et al*., 2022), highlighting a possible evolutionarily conserved mechanism of vector manipulation and dissemination.

## Concluding remarks

Each biological variable added to a multitrophic system increases its complexity and emphasizes the importance of understanding the holobiome involved over the different types of binary interactions that are usually studied, as this approach gets closer to mimicking what actually happens in nature and field crops. This review aimed to highlight important and emerging multitrophic interactions in sugarcane that impact pest and disease control programs. The understanding of the different variables that influence these complex biological systems are paramount for the development of new control strategies that are more environmental friendly and cost effective. Much of the knowledge generated from classical binary interaction studies cannot be translated to real-life conditions, in which myriad variables are added, influencing the expected results. As such, the complexity generated by all possible interactions occurring in one or more pathosystems is still difficult to define and study. Nevertheless, research focusing on these systems is bound to have a greater real-life impact and aid in the development of better strategies for improving the production of crops such as sugarcane.
